# Molecular Insights into Tumor Immunogenicity

**DOI:** 10.3390/cimb47080641

**Published:** 2025-08-11

**Authors:** Irini Doytchinova, Stanislav Sotirov, Ivan Dimitrov

**Affiliations:** Drug Design and Bioinformatics Lab, Faculty of Pharmacy, Medical University of Sofia, Dunav St. 2, 1000 Sofia, Bulgaria; 113660@students.mu-sofia.bg (S.S.); idimitrov@pharmfac.mu-sofia.bg (I.D.)

**Keywords:** HLA class I binders, T-cell epitopes, molecular dynamics simulations, immunogenicity, neoantigen prediction, vaccine design, TCR-based immunotherapies

## Abstract

Tumor immunogenicity depends on the ability of peptides to form stable and specific interactions with both HLA molecules and T-cell receptors (TCRs). While HLA binding is essential, not all HLA-binding peptides elicit T-cell responses. This study investigates the molecular features distinguishing immunogenic T-cell epitopes from non-immunogenic HLA binders. Two datasets of nonamer peptides—38 T-cell epitopes and 144 non-epitopes—were compiled and analyzed using sequence logo models and molecular dynamics (MD) simulations of TCR–peptide–HLA complexes. A comparative logo analysis revealed strong amino acid preferences at central positions (p4–p8) in T-cell epitopes and absences in non-epitopes. A representative epitope–non-epitope pair was selected for structural modeling and 100 ns MD simulations. The T-cell epitope formed a more stable complex with the TCR and exhibited greater flexibility, supporting an induced-fit recognition mechanism. It also established a broader and longer-lasting network of hydrogen bonds and π interactions across the residues at positions p4–p8. In contrast, the non-epitope engaged TCR at only two positions. These findings highlight the critical role of the peptide’s central region in TCR engagement and provide structural insights useful for neoantigen prediction, vaccine design, and TCR-based immunotherapies.

## 1. Introduction

The interaction between T-cell receptors (TCRs) and major histocompatibility complex (MHC) molecules plays a pivotal role in adaptive immunity, influencing immune recognition and response to pathogens and cancer [1,2]. T-cell epitopes are short peptide sequences derived from antigens that bind to MHC and are recognized by TCRs to initiate an immune response. Human MHC molecules are named human leukocyte antigens (HLA). Since antigen presentation relies on the binding of peptides to HLA molecules, all T-cell epitopes must inherently be HLA binders. However, not all peptides that bind to HLA molecules elicit a T-cell response, highlighting the distinction between HLA binding and T-cell epitope functionality. The ability of a peptide to bind an HLA molecule is a necessary but insufficient condition for T-cell activation. Other factors, such as peptide processing, stability within the HLA complex, and interaction with specific TCRs, determine immunogenicity [3]. Many peptides exhibit strong HLA binding but fail to trigger T-cell activation due to insufficient TCR recognition or lack of co-stimulatory signals [4]. Advances in immunoinformatics have helped predict HLA binding with high accuracy, yet experimental validation remains crucial to identifying true T-cell epitopes [5].

The specificity and cross-reactivity of TCRs are governed by the structural and biochemical properties of the peptide–MHC (pMHC) complex, which can significantly impact immune surveillance and immunotherapeutic applications [6,7]. A deeper understanding of these interactions is essential for advancing immunotherapy and vaccine design, particularly in the context of cancer and infectious diseases [8,9]. Recent studies have provided valuable insights into the structural and functional dynamics of TCR–peptide–MHC binding, shedding light on the mechanisms that govern antigen recognition, specificity, and cross-reactivity [10,11].

Coles et al. [12] explored the structural and functional diversity of TCRs binding to the same pMHC complex. By analyzing crystal structures and specificity profiles of three human TCRs targeting the NY-ESO-1157-165-HLA-A2 complex, the study highlighted two main findings: two TCRs recognized a common peptide feature but exhibited differences in peripheral tolerance, while a third TCR engaged the peptide in a flipped conformation, leading to a distinct specificity profile. These findings emphasize the role of TCR cross-reactivity in expanding immune recognition despite a limited TCR repertoire, providing insights relevant to TCR engineering for cancer immunotherapy [13,14].

Similarly, Smith et al. [15] investigated the impact of subtle modifications at MHC anchor residues on TCR recognition. Their study demonstrated that even structurally silent anchor modifications could significantly alter TCR binding affinity through allosteric effects that propagate via the MHC protein [16,17]. The observed receptor-dependent variations in sensitivity to these modifications have crucial implications for designing improved peptide vaccines and predicting neoantigen immunogenicity [18,19].

Further advancing this field, Du et al. [20] introduced the TRACeR-I platform, an innovative system for engineering peptide-targeting molecules with broad MHC I compatibility. This platform enables highly specific recognition of peptide antigens across multiple HLA alleles, overcoming limitations posed by HLA polymorphism [21,22]. Structural analysis revealed a unique antigen recognition mechanism, allowing single-residue specificity and enabling TRACeR-based bispecific T-cell engagers and chimeric antigen receptor (CAR) T cells to exhibit potent tumor cell killing in vitro [23,24]. While this approach presents exciting possibilities for immunotherapy and diagnostic applications, further in vivo validation is necessary to assess its clinical relevance.

These studies collectively enhance our understanding of TCR–peptide–MHC interactions and their implications for therapeutic development. As research continues to evolve, future efforts should focus on expanding investigations to diverse MHC alleles and antigenic peptides [25,26], exploring in vivo applications of engineered TCRs [27,28], and refining computational modeling to improve immune interaction predictions [29,30]. The insights gained from these studies hold promise for advancing personalized immunotherapy and next-generation immune-targeting therapeutics [31,32].

In this study, we investigate the subtle distinctions between T-cell epitopes and non-T-cell epitopes among HLA class I binders. To achieve this, we compile two separate sets of HLA class I nonamer binders: one consisting of confirmed T-cell epitopes and the other comprising non-T-cell epitopes. The amino acid frequencies at each position within the peptides are normalized for both sets. A comparative analysis of amino acid composition across peptide positions reveals several well-defined differences, particularly in the central region of the peptide structure. These findings provide valuable insights into the molecular features that distinguish immunogenic epitopes from non-immunogenic binders.

## 2. Datasets and Methods

### 2.1. Datasets

#### 2.1.1. Set of T-Cell Epitopes

Information on 212 immunogenic human tumor peptides was primarily obtained using the PubMed database’s ‘Similar Articles’ tool [33]. Immunogenicity was confirmed based on evidence from positive MHC binding assays and successful T-cell activation assays conducted in vivo in human subjects. To ensure accuracy and consistency, the extracted data were cross-verified with corresponding peptide records in the Immune Epitope Database (IEDB, San Diego, CA, USA) [34]. The dataset of T-cell epitopes is freely available for download at https://www.ddg-pharmfac.net/vaxijen3/tumordb/home/ (accessed on 20 April 2025) For the purposes of the current study, only nonamer peptides were selected, resulting in a final set of 38 T-cell epitopes.

#### 2.1.2. Set of Non-T-Cell Epitopes

A matched negative set comprising 212 non-immunogenic tumor peptides was compiled from the literature. These peptides were defined by the absence of a T-cell response despite exhibiting confirmed binding to MHC molecules. Therefore, the classification criteria for non-immunogenicity required positive results in MHC binding assays and negative outcomes in T-cell activation assays conducted in vivo in human subjects. The resulting dataset of non-T-cell epitopes is freely available at https://www.ddg-pharmfac.net/vaxijen3/tumordb/home/ (accessed on 20 April 2025). For the present study, only nonamer peptides were selected, yielding a final set of 144 non-T-cell epitopes.

#### 2.1.3. X-Ray Structure of TCR-Peptide-HLA Class I Complex

An X-ray crystal structure of a TCR–peptide–HLA class I complex was obtained from the Protein Data Bank (PDB ID: 6RPA) [12]. Missing regions were reconstructed, and hydrogen atoms were added. A mirror complex was generated by mutating the residues of the bound peptide to produce a structure containing a non-T-cell epitope. The structural stability of both complexes was evaluated using molecular dynamics simulations.

### 2.2. Logo Method

The frequency of each amino acid at a given nonamer position within a peptide set is normalized using the following formula:

$$X_{i}\left(norm\right)=\frac{\left(X_{i}-X_{mean}\right)}{\left(X_{max}-X_{min}\right)}$$

where *X_i_* represents the frequency of amino acid *i* at a specific position; *X_mean_* is the average frequency across all nine positions; and *X_max_* and *X_min_* are the maximum and minimum frequencies observed across all nine positions, respectively. This normalization scales the values to the range [−1, 1].

The resulting normalized data are arranged into a quantitative matrix (QM) of dimensions 9 positions × 20 amino acids. This matrix is referred to as a logo model [35,36], inspired by sequence logos—graphical representations of amino acid conservation in protein sequences—where each position is visualized as a stack of letters, with letter height corresponding to amino acid frequency [37].

### 2.3. Molecular Dynamics (MD) Simulations

The TCR–peptide–HLA class I complexes were placed within a truncated octahedral simulation box, solvated with TIP3P water. Sodium and chloride ions (NaCl) were added to achieve physiological ionic strength and to neutralize the overall charge of the complexes. The solvated systems underwent energy minimization for 5000 steps with harmonic restraints (3 kcal/mol·Å^2^), followed by a 1 ns heating phase from 0 to 310 K under constant volume, maintaining the same restraints. Then, the systems were equilibrated under constant pressure: first for 1 ns with restraints, then for 100 ns without restraints. Finally, production MD simulations were then carried out for 100 ns at a constant temperature (310 K) and pressure (1 bar), using a Langevin thermostat [38] and Berendsen barostat [39], respectively. All simulations employed the ff14SB force field [40] and periodic boundary conditions. A cutoff of 12.0 Å was applied to both van der Waals and electrostatic interactions, with long-range electrostatics beyond this threshold treated using the particle-mesh Ewald (PME) method [41]. Covalent bonds involving hydrogen atoms were constrained using the SHAKE algorithm [42] throughout the heating, equilibration, and production phases, enabling a 2 fs integration time step. These constraints were not applied during energy minimization. During production runs, simulation snapshots were saved every 1 ns, yielding 100 frames per trajectory for subsequent analysis. Three runs were performed for each complex using AMBER 18 [43], and trajectory analyses were conducted with cpptraj version 4.24.0 [44].

## 3. Results

### 3.1. Logo Model for T-Cell Epitopes

**Figure 1:**
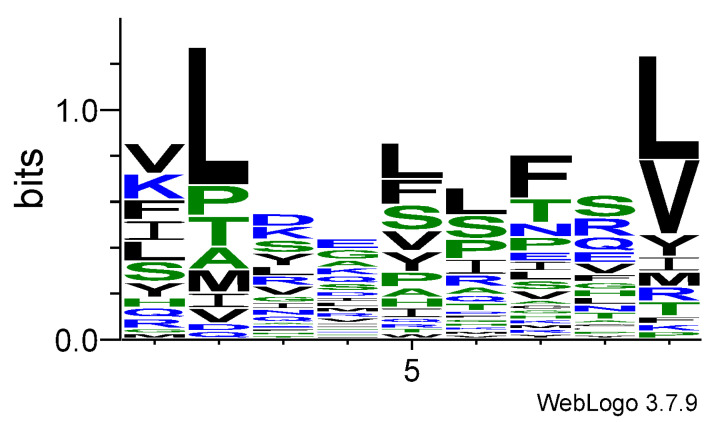
Sequence logo for T-cell epitopes.

**Figure 2:**
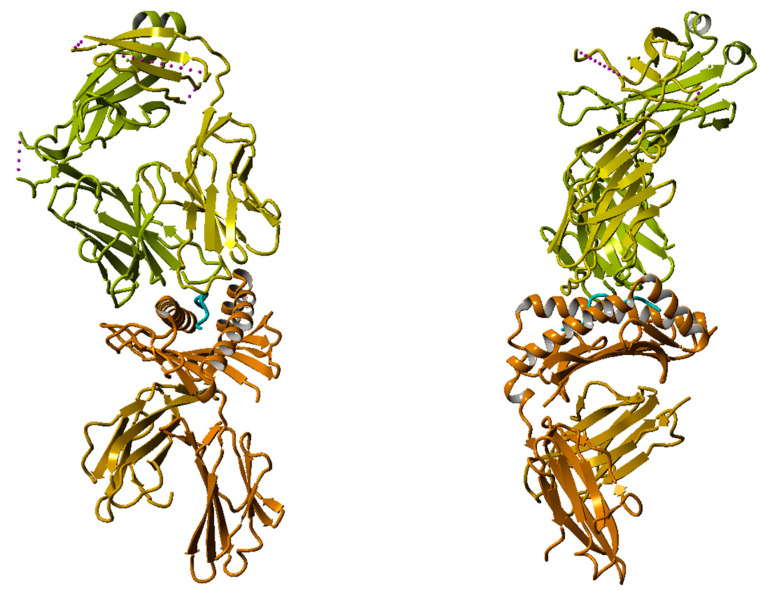
X-ray crystal structure of the HLA–peptide–TCR complex (PDB code: 6RPA). (**Left**): View across the peptide binding groove; (**right**): view along the peptide binding groove. The HLA molecule is shown in orange, β-microglobulin in gold, TCR chain D and E in yellow and light green, respectively, and the peptide in cyan.

The logo model for T-cell epitopes is presented in Table 1, and the corresponding sequence logo graph is given in Figure 1. Strong preferences exist at specific positions. As illustrated by the X-ray structure of the HLA–peptide–TCR complex (Figure 2; PDB code: 6RPA) [12], positions p2 and p9 serve as anchor residues within the HLA binding groove, while positions p4, p5, and p8 are primarily involved in TCR recognition. Residues at positions p1, p3, p6, and p7 are oriented tangentially, enabling potential interactions with both HLA and TCR molecules. A similar peptide conformation is consistently observed across most HLA–peptide–TCR complexes available in the Protein Data Bank (PDB) [45].

Leu has the highest positive coefficients at p2 (0.894) and p9 (0.672). Indeed, 18 T-cell epitopes in the set contain Leu at p2 and 14 at p9. These coefficients suggest that Leu is a strong anchor residue, likely contributing to MHC I binding. The second most frequent residue at p9 is Val with a coefficient of 0.450. It is available in 10 epitopes, reinforcing the importance of hydrophobic anchors at p9.

Val, followed by Lys, is the most frequent residue at p1. Phe, Ile, Leu, and Ser are also commonly observed at this position. At p3, Asp, Lys, Ser, and Tyr are preferred. Glu and Gly are the most frequent residues at the TCR-exposed p4. Leu, followed by Phe and Ser, is favored at p5. P6 is most often occupied by Leu and Ser, p7 by Phe and p8 by Ser. The moderate prevalence of hydrophilic residues at central positions suggests a potential role in TCR specificity. Conversely, the consistently negative scores for Cys, Trp, and Glu across nearly all positions indicate that these residues are generally poorly tolerated in T-cell epitopes.

### 3.2. Logo Model for Non-T-Cell Epitopes

**Figure 3:**
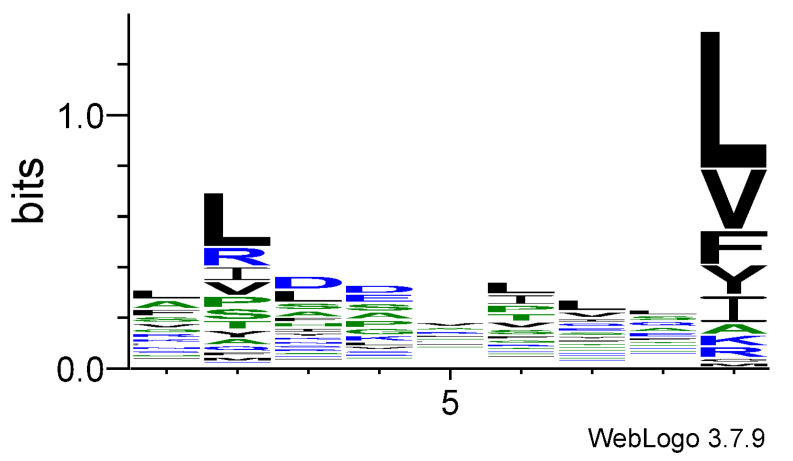
Sequence logo for non-T-cell epitopes.

The logo model for T-cell epitopes is presented in Table 2, and the corresponding sequence logo graph is given in Figure 3. In general, the amino acid preferences in non-T-cell epitopes are less distinct. The overall range of coefficients in the quantitative matrix (QM) is significantly narrower, reflecting weaker selective pressure for specific residues. While Leu remains moderately favored at the anchor positions p2 and p9, its preference is reduced compared to that observed in T-cell epitopes. In the central region of the peptide, the compressed preference profile suggests limited engagement by TCR. Leu shows mild enrichment at positions p1, p6, p7, and p8, whereas Asp is preferentially selected at p3 and p4.

### 3.3. Molecular Dynamics (MD) Simulations of HLA–Peptide–TCR Complexes Containing a T-Cell Epitope and a Non-T-Cell Epitope

To identify the structural differences between T-cell epitopes and non-T-cell epitopes, we performed a comparative analysis of nonamer peptides from both groups. Three pairs of closely related structural analogues were identified, each sharing four identical residues (Table 3). An X-ray of the crystal structure of the HLA–peptide–TCR complex is available only for the T-cell epitope SLLMWITQV (PDB ID: 6RPA) [12]. The complex containing the non-T-cell epitope GLLRVISGV was modeled by structural homology.

Both complexes underwent three independent 100 ns MD simulations, following the protocol outlined in the Datasets and Methods section. To capture a potential dissociation event, additional extended simulations of 1000 ns were performed for the complex containing the non-T-cell epitope. No dissociation was observed for the period of 1000 ns. Trajectory analyses revealed several distinct structural and dynamic differences between epitope-and non-epitope-containing complexes.

### 3.4. The T-Cell Epitope Forms a More Stable Complex with HLA and TCR

**Figure 4:**
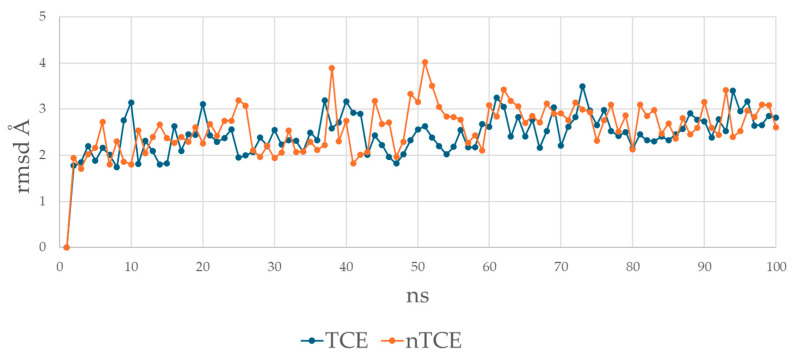
Average backbone RMSDs over 100 frames (100 ns) of the HLA–peptide–TCR complexes containing T-cell epitope SLLMWITQV (ink blue) and non-T-cell epitope GLLRVISGV (orange).

The average backbone RMSDs (root mean square deviations) of the HLA–peptide–TCR complexes were calculated as a function of time (Figure 4). The T-cell epitope forms a more stable complex, with an RMSD (avg) of 2.44 Å and with 10 extreme deviations higher than 3 Å compared to an RMSD (avg) of 2.58 Å with 21 extreme spikes for the non-T-cell epitope. The RMSD (max) reaches 3.50 Å at the 73rd ns for the T-cell epitope vs. 4.02 Å at the 51st ns for the non-T-cell epitope. The higher number of RMSD spikes suggests that the non-T-cell epitope introduces instability or fails to preserve native interactions during the simulation.

### 3.5. The T-Cell Epitope Is More Flexible in the Complex with HLA and TCR

**Figure 5:**
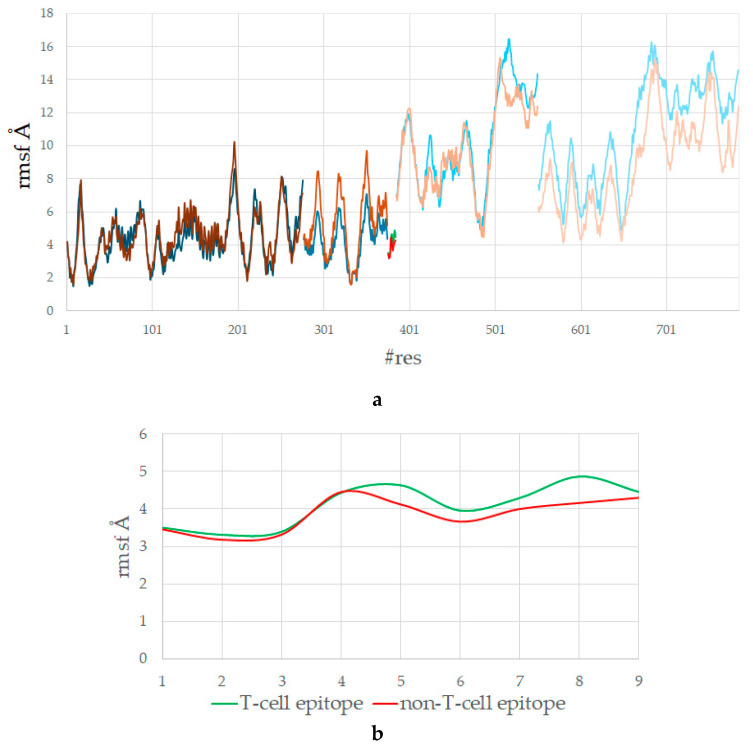
(**a**) Average RMSFs by residue of the HLA–peptide–TCR complexes containing T-cell epitope SLLMWITQV (blue shades) and non-T-cell epitope GLLRVISGV (brown shades). Residues 1–276 correspond to HLA protein, residues 277–375 to β-microglobulin, residues 376–384 to peptide (epitope given in green, non-epitope in red), residues 385–550 to TCRα, residues 551–785 to TCRβ. (**b**) Average RMSFs by residue of the bound peptide only.

The average RMSFs (root mean square fluctuations) of the HLA–peptide–TCR complexes were calculated per residue (Figure 5a). For more clarity, the average RMSFs by protein/peptide chain are given in Table 4.

Although the HLA–T-cell epitope–TCR complex is more stable than the complex containing a non-T-cell epitope, the former complex shows slightly higher overall flexibility: 7.568 Å vs. 7.039 Å. This observation might initially appear counterintuitive—given the need for stable HLA and TCR binding—but it can be functionally advantageous. Flexibility is due to biological readiness for TCR interaction, not instability. The T-cell epitopes engage diverse residues from TCR, which requires conformational flexibility to enable an induced-fit mechanism during TCR recognition and has been linked to increased immunogenicity [2,46].

Position-by-position analysis of the peptides reveals that the most pronounced differences in atomic flexibility occur at positions p5 and p8, with higher fluctuations observed in the T-cell epitope (Figure 5b). This can be attributed to the presence of the bulkier Trp residue at p5 in the epitope, which exhibits greater conformational mobility compared to the much smaller Val found at the same position in the non-epitope. A similar trend is seen at p8, where the long and flexible Glu in the epitope displays higher atomic fluctuations than the small, inert Gly residue in the non-epitope.

### 3.6. Residues from the Middle Part of the T-Cell Epitope Form Long-Lasting Hydrogen Bonds with TCR

**Figure 6:**
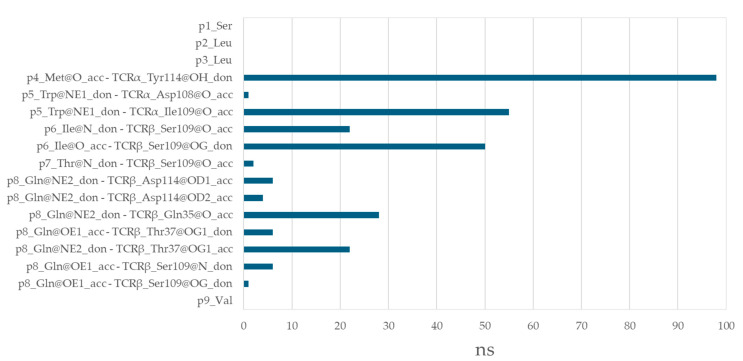
Hydrogen bonds between the T-cell epitope SLLMWITQV and TCR α and β chains.

The T-cell epitope SLLMWITQV forms a total of 13 hydrogen bonds with the TCR α and β chains, collectively lasting 301 ns (Figure 6). A significant proportion of these bonds exhibit remarkable stability, with 91% persisting for over 20 ns and 67% exceeding 50 ns. Met at p4 forms a particularly stable hydrogen bond of 98 ns with Tyr114 from TCRα. Trp at p5 engages in two interactions: one with Ile109 from TCRα for 55 ns and another with Asp108 for 1 ns. Ile at p6 interacts with Ser109 from TCRβ for a substantial 72 ns. For one-third of this duration, Ile acts as a hydrogen bond donor via its backbone nitrogen, while for the remaining two-thirds, it functions as an acceptor through its carbonyl oxygen. Thr at p7 forms a short-lived bond of 2 ns with Ser109. Gln at p8 interacts with Gln35 and Thr37 from TCRβ for 28 ns each. Additionally, Gln forms several transient hydrogen bonds (up to 10 ns) with Ser109 and Asp114 from TCRβ.

**Figure 7:**
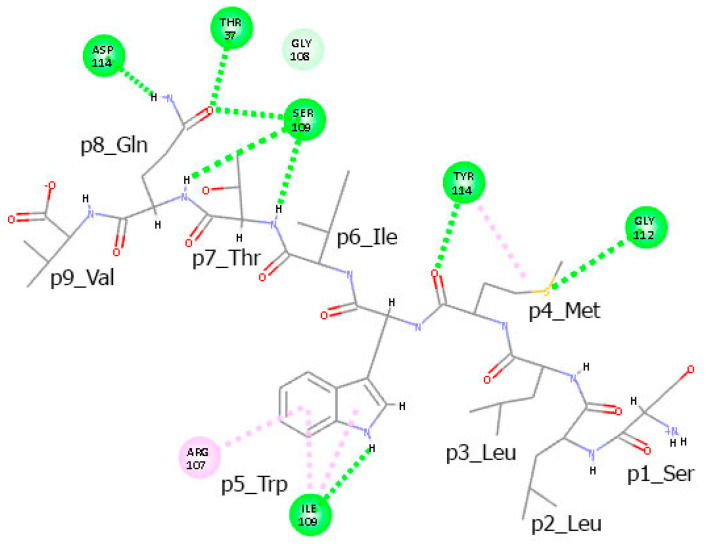
Intermolecular interactions between the T-cell epitope SLLMWITQV and the TCR α and β chains observed in the final conformation of the complex at the 100th ns. Hydrogen bonds are represented by green dashed lines, while π–alkyl and π–cation interactions are indicated by pink dots.

In addition to hydrogen bonds, several peptide residues participate in π–alkyl and π–cation interactions with the TCR chains. The lone electron pair of the sulfur atom in Met at p4 interacts with the π-electron cloud of Tyr114 from TCRα (Figure 7). Similarly, the π–electron cloud of the indole ring of Trp at p5 engages in π–alkyl interactions with the alkyl groups of Ile109 and in π–cation interactions with the positively charged Arg107, both from TCRα.

**Figure 8:**
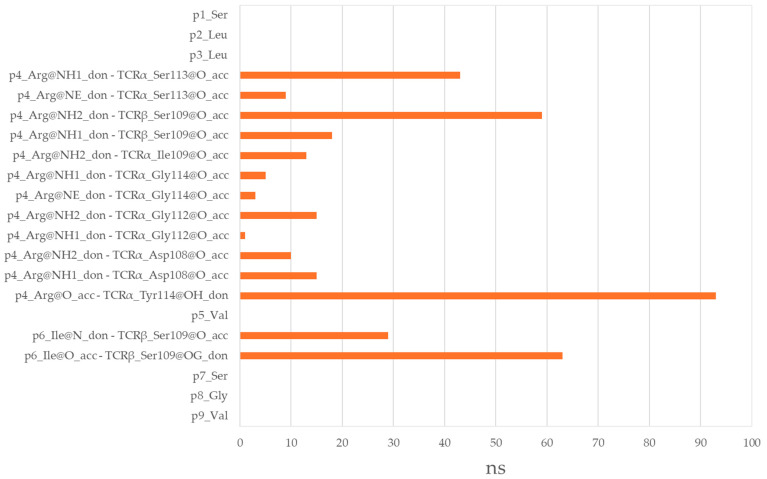
Hydrogen bonds between the non-T-cell epitope GLLRVISGV and TCR α and β chains.

Interestingly, the non-T-cell epitope GLLRVISGV forms a total of 14 hydrogen bonds with the TCR α and β chains, accumulating 376 ns of total interaction time (Figure 8). While a good portion of these bonds show stability (76% lasting over 20 ns and 57% over 50 ns), it should be noted that only two peptide residues, Arg at p4 and Ile at p6, are involved in these interactions. The TCR-exposed residues at p5, p7, and p8 remain uninvolved. Arg at p4 forms a stable hydrogen bond of 93 ns with Tyr114 from TCRα. Additionally, it forms hydrogen bonds with Ser109 from TCRβ for 77 ns and with Ser113, Asp108, Ile109, and Gly112 from TCRα for 52 ns, 25 ns, and 16 ns, respectively. Ile at p6 interacts with Ser109 from TCRβ for 92 ns. Here again, for one-third of this period, Ile acts as a hydrogen bond donor through its backbone nitrogen, and as an acceptor for the remaining two-thirds through its carbonyl oxygen.

**Figure 9:**
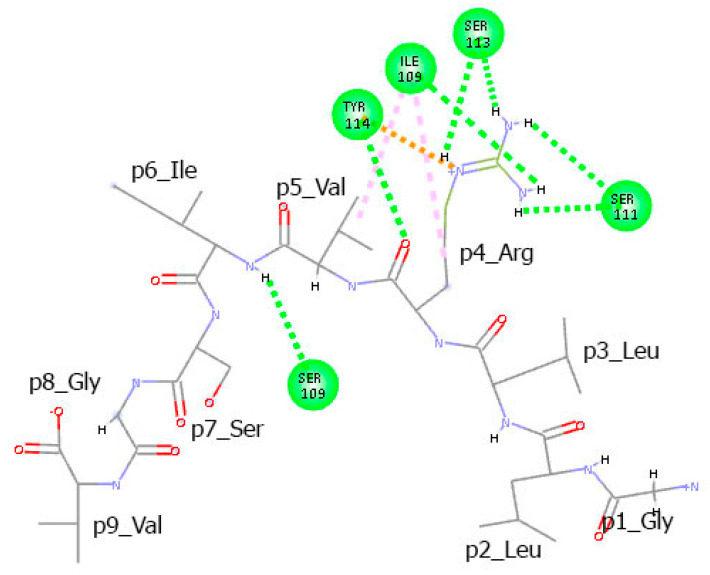
Intermolecular interactions between the non-T-cell epitope GLLRVISGV and the TCR α and β chains observed in the final conformation of the complex at the 100th ns. Hydrogen bonds are represented by green dashed lines, while alkyl and π–cation interactions are indicated by pink and orange dots, respectively.

The alkyl side chains of Arg at p4 and Val at p5 engage in hydrophobic interactions with Ile109 from TCRα (Figure 9). Additionally, the cationic group of Arg at p4 forms a π–cation interaction with Tyr114 from TCRα.

## 4. Discussion

HLA polymorphism is a fundamental pillar of human immunity. It plays a crucial role in the survival of the human species by enabling the immune system to recognize and respond to a vast array of pathogens. Throughout history, despite the emergence of deadly infectious diseases, no single pathogen has been able to eradicate the entire human population—proof of the protective diversity of the HLA system. This genetic variability ensures that different individuals present different sets of peptides to T cells, providing population-level resistance to infectious agents. One reason for that is the difference in the binding specificity. A peptide may not bind stably to a certain HLA allele but may bind strongly to another one. For example, the Gag-derived epitope SLYNTVATL is presented by HLA-A02:01 and induces strong CD8^+^ T-cell responses. However, individuals lacking HLA-A*02:01 may not mount a similar response unless they carry other alleles like HLA-B*57, which are associated with slower disease progression due to recognition of different HIV peptides [47].

Another reason is the TCR recognition differences. The conformation of the peptide in the binding groove differs across HLA alleles, altering TCR accessibility [7]. This can lead to differential T-cell responses. Even if a peptide binds to multiple alleles, variations in the peptide–HLA surface can affect TCR engagement and immune response. Some peptides are promiscuous epitopes, capable of binding and being presented by many HLA alleles, which enhances their likelihood of T-cell recognition across different individuals. Conversely, many epitopes are HLA-specific, and immunogenicity depends on both binding affinity and recognition.

Even in healthy cells, a small but steady percentage of proteins are synthesized incorrectly—possibly 5–15% under normal conditions. This rate increases under stress, aging, or disease conditions (especially in cancer) [48]. These proteins often misfold and are rapidly degraded via the MHC class I antigen presentation pathway, which presents endogenous (intracellular) peptides to CD8^+^ cytotoxic T cells [49]. The miscoded proteins are tagged with ubiquitin and degraded by the proteasome, producing short peptide fragments (8–12 amino acids). Peptides are transported from the cytosol into the endoplasmic reticulum (ER) via the TAP transporter (transporter associated with antigen processing). In the ER, peptides bind to the MHC class I molecules with the help of chaperone proteins (e.g., tapasin, calreticulin). Only peptides with high affinity and correct length are successfully loaded. The peptide–MHC I complexes are transported to the cell surface. CD8^+^ T cells recognize these complexes, potentially triggering an immune response if the peptide is seen as foreign.

In tumor cells, genetic instability increases the frequency of frameshift mutations, splicing errors, and translational infidelity [50]. These errors give rise to non-canonical peptides (neoantigens) that are absent in normal cells and can be recognized by the immune system [51]. These neoantigens are potential targets for cancer immunotherapy, like neoantigen vaccines and T-cell receptor (TCR)-engineered T cells [52,53].

T-cell recognition is a highly specific immune process determined by the interaction between a TCR and a peptide–MHC complex on the surface of antigen-presenting cells. The amino acid sequence and conformation of the peptide presented by MHC are key determinants of T-cell recognition. Even single amino acid substitutions can drastically alter recognition. MHC polymorphism (HLA in humans) determines which peptides are bound and presented and how the peptide–MHC is “seen” by different TCRs [54]. TCR recognizes both the MHC and the peptide. The stability and half-life of the peptide–MHC complex on the cell surface are crucial. More stable complexes tend to be more immunogenic [55]. Recognition by TCRs is influenced by the variable regions of the TCR (Vα and Vβ), e.g., the TCR repertoire diversity. Additionally, co-receptors (e.g., CD8 or CD4) stabilize the TCR–peptide–MHC interaction [1].

The middle part of a peptide plays a crucial and dominant role in determining the specificity and affinity of the interaction between the peptide–MHC complex and TCR. In the present study, we compared the amino acid frequencies at each peptide position between two sets of HLA class I binders: T-cell epitopes and non-T-cell epitopes. As expected, both groups showed similar preferences at anchor positions p2 and p9. However, distinct differences emerged in the central region of the peptides. T-cell epitopes showed notable preferences for specific residues in this region: Glu and Gly at p4, Leu, Phe, and Ser at p5, Leu and Ser at p6, Phe at p7, and Ser at p8. In contrast, the non-T-cell epitopes exhibited no clear preferences at these positions. To further investigate the functional consequences of these differences, we performed 100 ns molecular dynamics simulations of HLA–peptide–TCR complexes, one containing a known T-cell epitope and the other a non-epitope.

Both complexes remained stable throughout the simulation. However, the HLA–T-cell epitope–TCR exhibits slightly higher overall flexibility, reflecting the induced-fit mechanism during TCR recognition. Significant differences were observed in the interactions between the residues from the middle part of the peptide and the TCR when comparing the epitope- and non-epitope-containing complexes. The T-cell epitope SLLMWITQV formed an extensive network of long-lasting hydrogen bonds involving all residues from p4 to p8. Additionally, Met at p4 established π interactions with Tyr114 and Gly112 of the TCR, and Trp at p5 interacted with Arg107 and Ile109. In contrast, the non-T-cell epitope GLLRVISGV engaged the TCR via hydrogen bonds at only two positions: Arg at p4 and Ile at p6. Arg at p4 also formed π interactions with Ile109 and Tyr114, but the overall interaction network was sparse and less stable.

A residue-by-residue comparison between the T-cell epitope and the non-epitope reveals several notable distinctions in their interactions with the TCR. Both the Met at position p4 in the epitope and the Arg at the same position in the non-epitope form stable hydrogen bonds with Tyr114 from the TCRα chain. However, the Trp at p5 in the epitope—being a bulky aromatic residue—establishes a rich network of interactions, including a hydrogen bond and a π–alkyl interaction with Ile109, as well as a π–cation interaction with Arg107. In contrast, the corresponding Val at p5 in the non-epitope lacks the size, polarity, and aromatic character necessary for strong TCR engagement. Ile at p6, present in both peptides, forms hydrogen bonds with Ser109 from the TCRβ chain. At position p7, Thr in the epitope participates in a short-lived hydrogen bond with Ser109, whereas the corresponding Ser in the non-epitope remains non-interactive. At p8, Gln in the epitope engages in multiple interactions with Gln35, Thr37, Ser109, and Asp114 from the TCRβ chain. In contrast, the corresponding Gly in the non-epitope is too small and inert to participate in any detectable TCR contacts.

Collectively, these findings support the conclusion that bulky, polar, and functionally interactive residues in the central part of the peptide are critical for forming a stable complex with the TCR. The epitope SLLMWITQV displays the structural and biochemical features characteristic of strong T-cell epitopes, while GLLRVISGV lacks essential TCR contact residues and shows no structural evidence of immunogenicity—explaining its classification as a non-T-cell epitope.

A similar peptide profile was observed in our previous study [56]. A dataset comprising 38 epitopes and 183 non-epitopes, all binding to HLA-A3 supertype alleles, was analysed using a combination of Comparative Molecular Similarity Indices Analysis (CoMSIA) and Soft Independent Modeling of Class Analogy (SIMCA). The resulting models identified a strong preference for polar amino acids with high electron density and hydrogen-bonding capacity at central peptide positions. These residues are well-positioned to protrude from the MHC binding groove and interact effectively with TCR, thereby contributing to the immunogenic potential.

In conclusion, the central portion of a peptide presented by MHC is the “hot spot” for TCR recognition. Its sequence and structure directly govern the strength, specificity, and immunogenicity of the T-cell response. Therefore, when designing or studying T-cell epitopes (e.g., in cancer or vaccine research), careful attention must be paid to the middle residues of the peptide.

## Figures and Tables

**Table 1:** Quantitative matrix (logo model) for T-cell epitopes. The most frequent residues at each position are given in bold.

aas	p1	p2	p3	p4	p5	p6	p7	p8	p9
A (Ala)	−0.106	0.061	−0.050	0.061	0.006	0.006	−0.050	−0.050	−0.106
C (Cys)	−0.106	−0.106	−0.106	−0.050	−0.106	−0.106	−0.050	−0.050	−0.106
D (Asp)	−0.106	−0.050	**0.117**	0.006	−0.106	−0.050	−0.106	−0.106	−0.106
E (Glu)	−0.106	−0.106	−0.050	**0.117**	−0.106	−0.106	0.006	0.061	−0.106
F (Phe)	0.117	−0.106	−0.050	−0.050	0.172	−0.050	**0.394**	0.006	−0.050
G (Gly)	−0.050	−0.106	0.006	**0.117**	−0.106	−0.050	−0.050	0.006	−0.106
H (His)	0.006	−0.106	−0.050	−0.050	0.006	−0.050	−0.050	0.006	−0.106
I (Ile)	0.117	0.006	0.006	0.006	0.006	0.117	0.006	−0.050	0.006
K (Lys)	0.172	−0.106	**0.117**	0.061	−0.106	−0.050	−0.050	−0.106	−0.050
L (Leu)	0.117	**0.894**	0.061	0.006	**0.283**	**0.283**	0.006	0.006	**0.672**
M (Met)	−0.050	0.061	−0.106	0.006	−0.106	−0.050	−0.050	−0.106	0.006
N (Asn)	−0.106	−0.106	0.006	−0.050	−0.106	−0.106	0.061	0.006	−0.106
P (Pro)	−0.106	0.117	−0.106	−0.050	0.061	0.172	0.061	−0.050	−0.050
Q (Gln)	0.006	−0.050	0.006	0.061	−0.050	0.006	−0.106	0.117	−0.106
R (Arg)	0.006	−0.106	0.061	0.006	−0.050	0.061	−0.050	0.172	0.006
S (Ser)	0.117	−0.106	**0.117**	0.061	0.172	0.228	0.006	**0.228**	−0.106
T (Thr)	−0.106	0.117	−0.050	−0.106	−0.050	0.006	0.172	0.006	0.006
V (Val)	**0.228**	0.006	0.061	0.006	0.117	−0.050	0.006	0.061	0.450
W (Trp)	−0.106	−0.106	−0.106	−0.106	−0.050	−0.106	−0.106	−0.106	−0.106
Y (Tyr)	0.061	−0.106	**0.117**	−0.050	0.117	−0.106	−0.050	−0.050	0.061

**Table 2:** Quantitative matrix (logo model) for non-T-cell epitopes.

aas	p1	p2	p3	p4	p5	p6	p7	p8	p9
A (Ala)	0.170	0.003	0.093	0.106	0.106	0.003	0.029	0.119	0.003
C (Cys)	−0.151	−0.163	−0.163	−0.138	−0.112	−0.138	−0.151	−0.151	−0.138
D (Asp)	−0.087	−0.074	0.272	0.170	−0.048	−0.074	−0.061	−0.087	−0.138
E (Glu)	−0.061	−0.048	0.054	0.183	0.003	−0.087	0.029	0.080	−0.112
F (Phe)	0.080	−0.099	−0.048	−0.087	−0.022	−0.048	−0.035	−0.010	0.067
G (Gly)	0.042	−0.061	0.016	0.131	0.080	0.080	0.003	0.042	−0.087
H (His)	−0.074	−0.112	−0.022	−0.074	−0.035	−0.087	−0.035	−0.035	−0.138
I (Ile)	−0.048	0.029	−0.022	−0.125	−0.035	0.080	0.003	−0.074	0.016
K (Lys)	−0.048	−0.151	−0.074	0.003	−0.022	−0.01	0.003	0.016	0.003
L (Leu)	0.375	0.696	0.247	0.042	0.067	0.234	0.311	0.183	0.837
M (Met)	−0.074	−0.074	−0.087	−0.125	−0.112	−0.112	−0.087	−0.099	−0.112
N (Asn)	−0.061	−0.151	−0.061	−0.099	−0.022	−0.061	−0.087	−0.074	−0.125
P (Pro)	−0.112	0.029	−0.061	0.119	0.054	0.183	0.029	−0.022	−0.138
Q (Gln)	−0.035	−0.035	0.003	0.016	−0.022	−0.061	0.042	0.093	−0.112
R (Arg)	0.093	0.144	−0.010	0.042	0.131	−0.010	−0.035	−0.022	−0.048
S (Ser)	0.144	0.106	0.106	0.119	0.054	0.093	0.029	0.131	−0.074
T (Thr)	−0.035	0.042	−0.087	−0.061	−0.010	0.119	0.003	0.106	−0.010
V (Val)	0.080	0.067	−0.061	0.016	0.170	0.106	0.170	0.016	0.260
W (Trp)	−0.138	−0.138	−0.099	−0.138	−0.112	−0.112	−0.112	−0.138	−0.151
Y (Tyr)	−0.061	−0.010	0.003	−0.099	−0.112	−0.099	−0.048	−0.074	0.196

**Table 3:** Pairs of structurally related T-cell and non-T-cell epitopes among the studied peptides. The identical residues are given in bold.

T-Cell Epitopes	Non-T-Cell Epitopes	Identical Residues	X-Ray Structures PDB Id
S**LL**MW**I**TQ**V**	G**LL**RV**I**SG**V**	4	6RPA, 6RPB, 6RP9 [12]
VTIG**PRL**L**L**	LPVS**PRL**Q**L**	4	-
R**A**TVA**PR**S**L**	S**A**YGE**PR**K**L**	4	-

**Table 4:** RMSFs by protein/peptide chain of the HLA–peptide–TCR complexes containing T-cell epitope and non-T-cell epitope.

RMSF (Avg)	T-Cell Epitope SLLMWITQV	Non-T-Cell Epitope GLLRVISGV
HLA	4.378	4.506
peptide (overall)	4.055	3.798
p1	3.505	3.463
p2	3.314	3.189
p3	3.394	3.323
p4	4.441	4.452
p5	4.644	4.125
p6	3.963	3.666
p7	4.303	4.004
p8	4.879	4.163
p9	4.464	4.302
TCRα	10.340	9.883
TCRβ	10.890	8.847
Total	7.568	7.039

## Data Availability

Data is contained within the article.

## Abbreviations

The following abbreviations are used in this manuscript:

HLA	Human leukocyte antigen
MD	Molecular dynamics
MHC	Major histocompatibility complex
nTCE	Non-T-cell epitope
pMHC	Peptide–MHC complex
RMSD	Root mean square deviations
QM	Quantitative matrix
RMSF	Root mean square fluctuations
TCE	T-cell epitope
TCR	T-cell receptor
